# Supplementary material for: Complex Relationships Between Homologous Recombination Deficiency (HRD) Score and Mutational Status of Homologous Recombination Repair (HRR) Genes in Prostate Carcinomas
Source: Int J Mol Sci. 2025 Dec 8;26(24):11851. doi: 10.3390/ijms262411851 (PMC12732970; doi:10.3390/ijms262411851)
Supplement: Supplementary file 1 [file ijms-26-11851-s001.zip › Supplementary Tables 2 and 3.pdf]

Supplementary Table S2. Characteristics of mutations in various HRR genes

Gene	Number of mutations in a particular gene (percentage of the total 262 mutations)	Number of cases with mutations (percentage of the total 216 cases with HRR mutations)	Share of cases with germline mutations, %	Share of cases with LOH* or double mutations, %	Share of cases with mutations in other HRR genes, %
BRCA2	45 (17.2%)	42 (19.4%)	29/38 (76.3%)	16/23 (69.6%)	6/42 (14.3%)
ATM	48 (18.3%)	42 (19.4%)	17/40 (42.5%)	16/18 (88.9%)	7/42 (16.7%)
CDK12	46 (17.6%)	30 (13.9%)	0/30 (0%)	16/30 (53.3%)	2/30 (6.7%)
CHEK2	26 (9.9%)	26 (12.0%)	25/26 (96.2%)	4/21 (19.0%)	7/26 (26.9%)
NBN	18 (6.9%)	15 (6.9%)	12/15 (80.0%)	3/12 (25.0%)	1/15 (6.7%)
FANCM	12 (4.6%)	12 (5.6%)	11/12 (91.7%)	0/7 (0%)	1/12 (8.3%)
FANCC	9 (3.4%)	9 (4.2%)	7/9 (77.8%)	0/4 (0%)	1/9 (11.1%)
BRCA1	9 (3.4%)	8 (3.7%)	8/8 (100%)	3/8 (37.5%)	1/8 (12.5%)
PALB2	6 (2.3%)	6 (2.8%)	6/6 (100%)	3/5 (60.0%)	1/6 (16.7%)
BLM	6 (2.3%)	6 (2.8%)	6/6 (100%)	0/5 (0%)	0/6 (0%)
RAD54L	5 (1.9%)	5 (2.3%)	4/5 (80.0%)	0/2 (0%)	1/5 (20.0%)
FANCI	5 (1.9%)	5 (2.3%)	5/5 (100%)	1/4 (25.0%)	1/5 (20.0%)
BRIP1	4 (1.5%)	4 (1.9%)	3/4 (75.0%)	0/3 (0%)	1/4 (25.0%)
FANCA	4 (1.5%)	4 (1.9%)	4/4 (100%)	0/3 (0%)	1/4 (25.0%)
BARD1	3 (1.1%)	3 (1.4%)	2/3 (66.7%)	nd	0/3 (0%)
MRE11	2 (0.8%)	2 (0.9%)	2/2 (100%)	0/2 (0%)	0/2 (0%)
RAD50	2 (0.8%)	2 (0.9%)	1/2 (50.0%)	0/1 (0%)	2/2 (100%)
Other	12 (4.6%)	12 (5.6%)	3/12 (25.0%)	0/6 (0%)	0/12 (0%)

*LOH in corresponding tumors was evaluated in cases with germline variants

Supplementary Table S3. Frequency of HRD score≥42 in cases with and without HRR mutations

Mutated genes	Frequency of HRD score ≥42	Median HRD score	Significant difference (p<0.01) from cases with other mutations
Any HRR gene	23/120 (19.2%)	16	WT
BRCA2	10/20 (50%)	41.5	BLM, CHEK2, WT
ATM	4/27 (14.8%)	24.0	CHEK2, WT
CDK12	1/13 (7.7%)	17.5	CHEK2
CHEK2	0/11 (0%)	6.0	ATM, BRCA2, BRIP1,CDK12
NBN	2/7 (28.6%)	9.0	
FANCM	1/6 (16.7%)	8.0	
FANCC	0/3 (0%)	10.0	
BRCA1	1/4 (25.0%)	13.0	
PALB2	2/4 (50.0%)	44.0	
BLM	0/4 (0%)	1.5	BRCA2
RAD54L	0/2 (0%)	8.0	
FANCI	0/2 (0%)	5.0	
BRIP1	2/3 (66.7%)	51.0	CHEK2
FANCA	0/3 (0%)	7.0	
BARD1	0/1 (0%)	14.0	
MRE11	0/2 (0%)	7.0	
Multiple	0/3 (0%)	17.0	
Other	0/6 (0%)	15.5	
WT	29/560 (5.2%)	10.0	ATM, BRCA2, any HRR gene
