# Supplementary figures and images for: Complex Relationships Between Homologous Recombination Deficiency (HRD) Score and Mutational Status of Homologous Recombination Repair (HRR) Genes in Prostate Carcinomas
Source: Int J Mol Sci. 2025 Dec 8;26(24):11851. doi: 10.3390/ijms262411851 (PMC12732970; doi:10.3390/ijms262411851)

**A**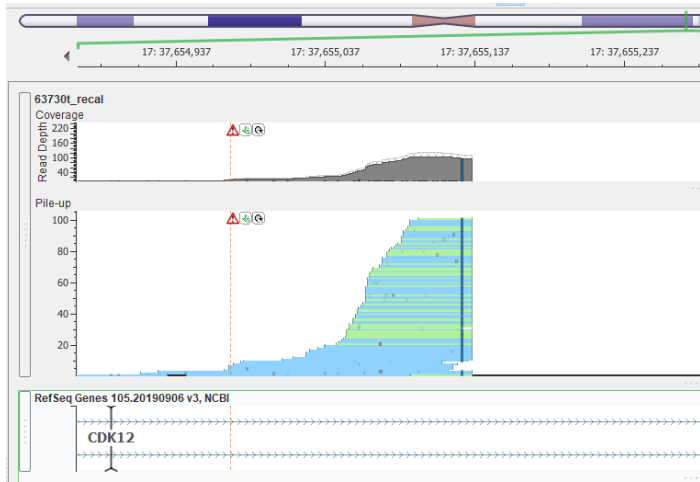

CDK12, intron 5

**B**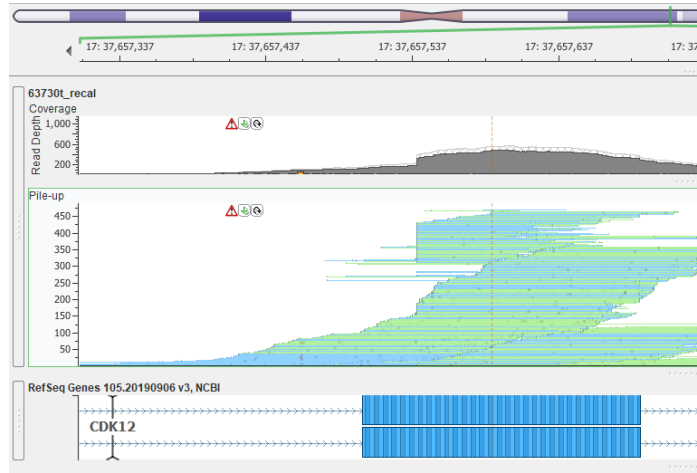

CDK12, exon 6

**C**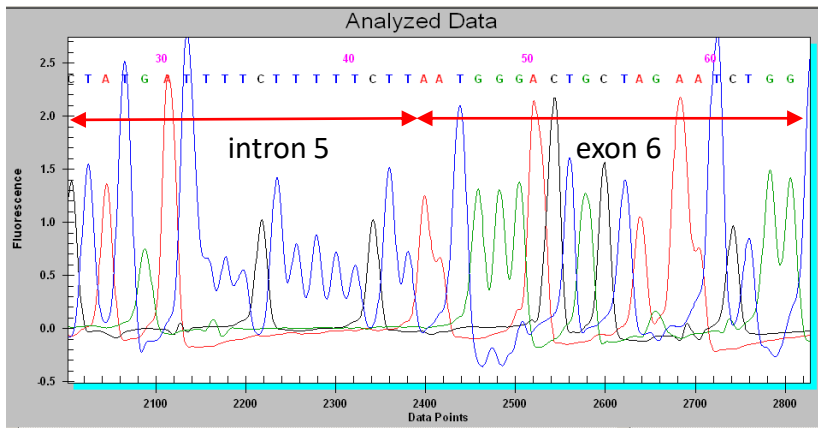

NM\_016507.4(CDK12)c.2420-2368\_2459del

Supplement: Supplementary file 1 [file ijms-26-11851-s001.zip › Supplementary Figure S2.pdf]

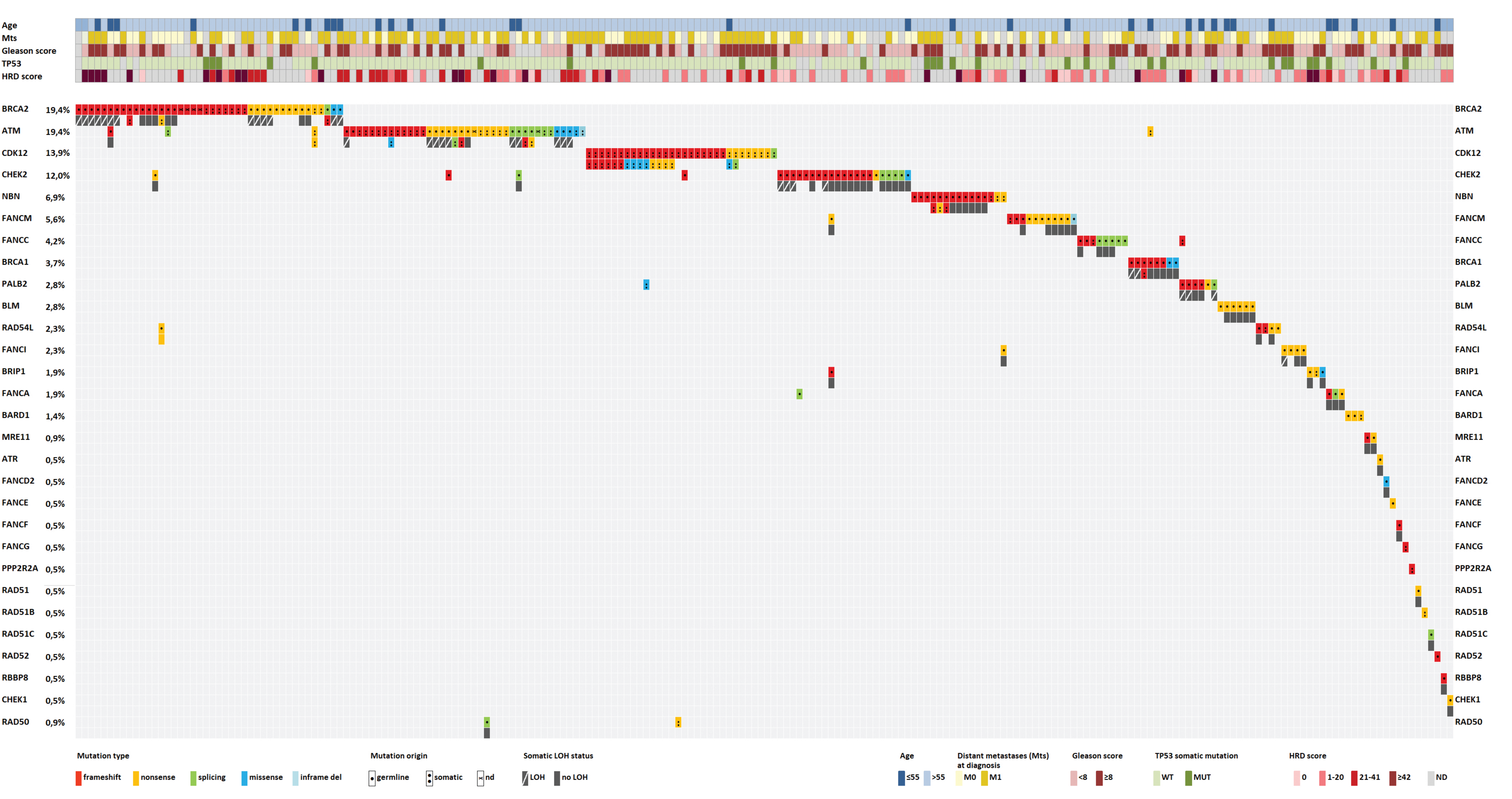

Supplement: Supplementary file 1 [file ijms-26-11851-s001.zip › Supplementary Figure S1.pdf]
